# Shoulder Tuberculosis: Management of a Posteriori Diagnosis After a Reverse Prosthesis Implantation

**DOI:** 10.1155/crdi/8378621

**Published:** 2025-06-28

**Authors:** Giordano G., Lourtet-Hascoët J., Martin Blondel G., Bonnet E.

**Affiliations:** ^1^Orthopedic-Traumatology Department, Joseph Ducuing Hospital, Toulouse, France; ^2^Microbiology Unit, Joseph Ducuing Hospital/Pasteur Clinic, Toulouse, France; ^3^Infection Disease Department, CHU Purpan, Toulouse, France; ^4^Mobile Infectiologist-Microbiology Team, Joseph Ducuing Hospital, Toulouse, France

**Keywords:** *Mycobacterium tuberculosis*, periprosthetic infection, reverse shoulder arthroplasty

## Abstract

We report a rare case of reverse shoulder prosthesis implantation in a patient retrospectively showing a chronic tuberculous osteoarthritis and meningitis. In this atypical presentation, the patient presented only a chronic shoulder pain, with no clinical or imaging sign of tuberculosis. After the implantation of a reverse shoulder prosthesis, the patient presented a reactivation of a tuberculosis causing a meningitis. The diagnosis of shoulder osteoarthritis caused by *M. tuberculosis* was confirmed retrospectively on deep perioperative samples by standard culture, PCR, and positive pathology. The management of the patient consisted in 12 months of a medical antituberculosis therapy and showed a favorable outcome.

## 1. Introduction

Bones infections represent only 3% of tuberculosis cases, and shoulder localization accounts for 1% of tuberculous osteoarthritis [1]. Some risk factors have been identified for bone infections caused by *M. tuberculosis*, including older age, immunosuppression (cancer and HIV infection), or steroid administration. Early manifestations are not typical with poor clinical signs and rare component loosening. Diagnosis is often performed in advanced phases, and complications such as bone damages and poor functional outcome are commonly reported [1].

In our case, the presentation is exceptional as evidenced by the analysis of the literature mainly composed of case reports. This article reports a reverse shoulder prosthesis implantation in a patient presenting a chronic tuberculous osteoarthritis, rarely reported with a retrospective diagnosis, after this implantation. It illustrates the challenges of the management of chronic osteoarticular infections in the context of a postimplantation diagnosis of infection, especially tuberculosis.

## 2. Case

A 73-year-old right-handed female, former nurse, presented with functional impairment and debilitating pain in her right shoulder. The symptoms evolved for 4 years, with a more present functional impairment over the past year. At the first consultation, no history of infection or tuberculosis during youth was reported. The infectious disease specialist questioned the patient on his medical history and treatments and did not report any previous history of infection. The overall history and symptoms were consistent with a diagnosis of glenohumeral osteoarthritis related to rheumatoid arthritis.

**Figure 1:**
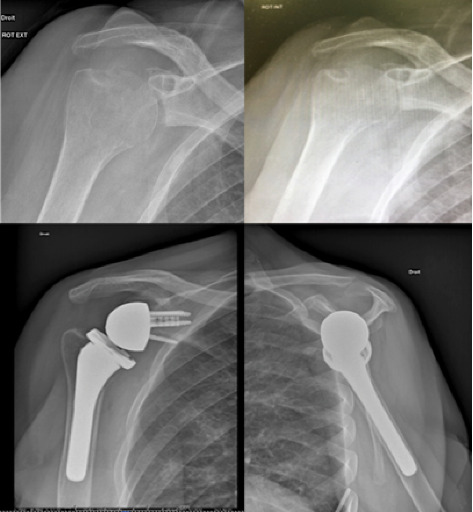
X-ray images before and after surgery.

Symptoms have been unsuccessfully treated for 3 years with methotrexate 10 mg/week. Clinical examination revealed an ankylosed shoulder with a pain score of seven, hand activity level of two, mobility at four, and muscle strength rated at zero. There was atrophy of the rotator cuff, particularly the infraspinatus muscle. The constant score was assessed at six preoperatively. Radiographically (Figure 1, upper images), the humeral head exhibited joint space narrowing without osteophytes. Osteolysis was observed on the cranial part of the humeral head near the greater tuberosity, with a sclerotic rim indicating a relatively slow process of joint degradation. Glenoid wear appeared as type A2 according to Walch classification.

These atypical radiological findings in a glenohumeral osteoarthritis prompted further investigation. A CT scan revealed signs of glenohumeral arthritis without infectious context. Bone density was significantly reduced, and atrophy of the rotator cuff, mainly affecting the supraspinatus and subscapularis muscles, was noted. Calcifications or bone fragments were found in the inferior recess, along with a detached bone fragment measuring 1.7 cm, constituting a true fracture. There was no evident joint effusion or subacromial–subdeltoid bursa effusion. MRI did not reveal any signs of infection. A cytobacteriological aspiration was performed, and the liquid aspect was clear. Cytology showed 35 leucocytes per mm^3^, the direct examination was negative. Cultures revealed no growth on solid and liquid media, under aerobic and anaerobic atmosphere after 15 days of standard culture. The electromyography remained negative. Following this evaluation, reverse shoulder arthroplasty was indicated after a multidisciplinary meeting. Surgery, performed via the deltopectoral approach, involved arthrolysis with the implantation of an Equinoxe prosthesis with a 4-mm augmented glenoid. The intraoperative macroscopic appearance showed no florid synovitis or suspicious intra-articular fluid. Glenoid losses involved the inferior rim and the center of the glenoid, with the bone appearing unremarkable. Systematic intraoperative samples were collected for cytobacteriological (including mycobacterial search) and histopathological analyses. The patient had uneventful immediate postoperative recovery. Her local and general condition remained uncomplicated. Imaging showed no anomaly (Figure 1, lower images). She was discharged home 5 days after the procedure.

Fifteen days after surgery, she was admitted to the hospital's emergency department with a fever of 38.5°C and a general state of malaise. There was no deterioration at the surgical site, and the joint was not painful. Frontal headaches and diplopia suggested meningitis. The patient was transferred to the intensive care unit. Blood cultures were negative, retrospectively, and MRI confirmed a peri-mesencephalic meningitis. A lumbar puncture revealed cerebrospinal fluid hypoglycorrhachia at 1 mmol/L, hypochloremia at 109, hyperproteinorachia at 1.9 g/L, white blood cells at 175/mm^2^, of which 90% were lymphocytes. Specific PCR for *Mycobacterium tuberculosis* in the cerebrospinal fluid was positive. Chest radiography was performed but no visible lesion was observed. Radiographic assessment of the shoulder was normal; a shoulder ultrasound showed no fluid. Histopathological examination of intraoperative samples, available 15 days after prosthesis placement, revealed epithelioid granulomatous lesions. Deep sample cultures showed *M. tuberculosis* susceptible to all tested antituberculosis drugs. Cultures from cerebrospinal fluid were also positive to *M. tuberculosis*. The diagnosis of the acute episode was therefore tuberculous meningoencephalitis.

The patient presented with bifocal involvement, with osteoarthritis of the shoulder diagnosed retrospectively. Quadruple antituberculosis therapy with Rifater (rifampicin, isoniazid, and pyranizamid 120/50/300 [6 tablets/day], for 2 months was initiated within 24 h, along with dexambutol 1.5 g/day, followed by a dual therapy with isoniazid and rifampicin [Rifinah] for a duration of 12 months). A follow-up PET scan was performed at 1 year, showing moderate hyperfixation limited to the anterior part of the shoulder joint capsule without bone involvement. Clinical evolution of the patient is favorable with an unpainful shoulder, and mobility is satisfactory (flexion 150°, abduction 90°, external rotation 40°, and internal rotation 10°). The patient's satisfaction score is 9. The constant score is 67. Radiographic and scintigraphy assessments are normal.

## 3. Discussion

The glenohumeral localization of tuberculosis is very rare according to analysis of the literature which is limited to few reported cases [2–10].  Table 1 summarizes the case reports with arthroplasties showing variable situations: arthritis treated by lavage, resection-arthroplasty, late infections on existing arthroplasties, chronic arthritis treated by arthroplasty [2–6, 8–10]. The case presented here corresponds to the latter scenario with a particularity: the diagnosis of shoulder tuberculosis was made retrospectively.  Table 1 collects the different cases of tuberculous infection with the presence of an arthroplasty. While *M. tuberculosis* is the predominant species, *Mycobacterium bovis* and *Mycobacterium conceptionense* may also be involved [2, 10].

Li et al. reported a case of late infection on prosthesis with *M. tuberculosis* in a 74-year-old patient undergoing chemotherapy for intestinal cancer, bearing a Neer humeral prosthesis for several years. In the presence of pain, moderate fever, and spontaneous fistula, two successive lavages were performed without success. The persistence of a fistula and positive samples for *M. tuberculosis* led to the removal of the prosthesis and placement of a spacer left permanently [6].

Amouyel et al. reported the case of a 73-year-old Portuguese patient with unexplained neutropenia following the implantation of a total reverse shoulder prosthesis on the right side for eccentric omarthrosis. Two months postoperatively, a peri-prosthetic infection was diagnosed with a purulent fistula observed. Poor evolution led to a puncture, which led to identification of *M. bovis* by PCR. The assessment revealed polyvisceral involvement, and implant removal with definitive arthroplasty resection was performed [2].

Ha et al. emphasize the diagnostic challenges in this localization and the nonspecific nature of the signs. None of the patients in their series presented with concomitant pulmonary tuberculosis, which is also the case with our patient [11]. Furthermore, at the first medical examination, no history of tuberculosis or any infection or pulmonary sign was reported, and no previous antibiotic treatment was mentioned in the medical record. Clinical presentation and radiological findings were also atypical when considering the diagnosis of omarthrosis. More generally, a careful review of clinical and imaging signs is constantly required in arthropathy indications. Clinical and/or radiological atypical features should prompt consideration of a low-grade evolving infection, notably tuberculosis. The exceptional nature of the glenohumeral localization partly explains why we did not pursue further specific preoperative investigations.

In situations of negative results or discrepancies in preoperative assessments, two types of complementary second-line examinations should be considered:

- Arthroscopic biopsies for cytobacteriological and histopathological purposes. In our practice, these are only performed in cases of persistent doubt with noncontributory first-line assessments (including joint aspiration). This was not argued for this patient.
- In atypical clinical presentations, the search for mycobacteria could be proposed systematically.

Our postoperative microbiological follow-up includes real-time reporting of positive results. This allows for prompt adaptation of antibiotic treatment. This protocol could not function since the cultures grew on the fifteenth day, coinciding with the neurological decompensation. This is one of the two reported cases in the literature of the implantation of a reverse shoulder prosthesis in chronic tuberculous arthritis [9]. The prior diagnosis of chronic tuberculous arthritis would probably not have motivated the same decision; a two-stage treatment with a long interval would have been chosen. Surgical management is not consensual in the literature, as prosthesis change or DAIR are described [2, 3, 6, 10]. The risk of treatment failure is high regarding to the localization, the difficulty of diagnosis, and of treatment of mycobacteria in bones. Therefore, final removal of the implant is reported in some cases [4, 7, 8].

Nevertheless, considering the clinical course and functional outcome at 3 years postsurgery, it seems possible to propose management with only appropriate antibiotic treatment, for patients with an unexpected diagnosis of chronic tuberculous arthritis. Our observation is in accordance with Spinner et al.'s proposals [12]. In the immediate aftermath of the neurological episode, a collective decision of surgical abstention armed surveillance concerning the implanted shoulder was made. Some studies reported low adherence and biofilm of mycobacteria on implant surface compared to other bacteria such as staphylococci [11]. These results may also favor a successful treatment with antituberculosis therapy only [12].

## 4. Conclusion

In this rare case, we report the implantation of a reverse shoulder prosthesis in a patient with a glenohumeral osteoarthritis, retrospectively showing chronic tuberculous osteoarthritis. This case illustrates diagnostic challenges and management of these delayed infections. Articular involvement with atypical presentations appears to benefit from arthroscopic biopsy with systematic search for mycobacteria. The question arises here regarding the management of postoperative diagnoses of peri-prosthetic joint infections.

## Figures and Tables

**Table 1:** Review of cases with mycobacteria infections in shoulder arthroplasties.

Meert et al. 2023 [3]	Disseminated tuberculosis	Case report	Reverse shoulder arthroplasty	*Mycobacterium tuberculosis*	Implant removal
Amouyel et al. 2019 [2]	Chronic arthritis	Case report	Reverse shoulder arthroplasty	*Mycobacterium tuberculosis*	Reverse shoulder arthroplasty
Uhel et al. 2018 [10]	/	Case report	Shoulder arthroplasty	*Mycobacterium tuberculosis*	Definitive resection arthroplasty
Langlois et al. 2016 [5]	Postoperative diagnosis 2 months and half	Case report	Reverse shoulder arthroplasty	*Mycobacterium bovis*	DAIR failure Definitive resection arthroplasty
Kim et al. 2015 [4]	Late infection	Case report	Reverse shoulder arthroplasty	*Mycobacterium tuberculosis*	Implant removal
Li et al. 2012 [6]	Chronic arthritis	Case report	Reverse shoulder arthroplasty	*Mycobacterium conceptionense*	Debridement J7
Lederman et al. 2011 [8]	Late infection	Case report	Hemiarthroplasty	*Mycobacterium tuberculosis*	Definitive spacer
Hattrup et al. 2008 [9]	Late infection	Case report	Humeral head replacement	*Mycobacterium tuberculosis*	Reverse shoulder arthroplasty refused by the patient

## Data Availability

Data sharing is not applicable to this article as no new data were created or analyzed in this study.
